# The prevalence of gene mutations in homologous recombination repair pathways in Japanese patients with metastatic castration‐resistant prostate cancer in real‐world clinical practice: The multi‐institutional observational ZENSHIN study

**DOI:** 10.1002/cam4.5333

**Published:** 2022-11-10

**Authors:** Hiroji Uemura, Mototsugu Oya, Toshiyuki Kamoto, Mikio Sugimoto, Kenta Shinozaki, Kiyomi Morita, Ryo Koto, Mai Takahashi, Masahiro Nii, Eisei Shin, Norio Nonomura

**Affiliations:** ^1^ Department of Urology and Renal Transplantation Yokohama City University Medical Center Yokohama City Japan; ^2^ Department of Urology Keio University School of Medicine Tokyo Japan; ^3^ Department of Urology, Faculty of Medicine Miyazaki University Miyazaki Japan; ^4^ Department of Urology, Faculty of Medicine Kagawa University Kagawa Japan; ^5^ AstraZeneca K.K Osaka Japan; ^6^ Department of Urology Osaka University Graduate School of Medicine Osaka Japan

**Keywords:** *BRCA*, homologous recombination repair, metastatic castration‐resistance prostate cancer, survival

## Abstract

**Background:**

Metastatic castration‐resistant prostate cancer (mCRPC) is a genetically heterogeneous disease with a poor prognosis. The prevalence of mutations in homologous recombination repair (HRR) pathway genes, including *BRCA1/2*, as well as treatment patterns and clinical outcomes, are not well characterized among Japanese men with mCRPC.

**Methods:**

This multicenter, noninterventional cohort study enrolled Japanese men with mCRPC from 24 institutions between 2014 and 2018. Mutations in the 15 HRR‐related genes were assessed using archival primary or metastatic tumor samples. Patterns of sequential therapies for mCRPC were investigated. Patients were followed up for survival evaluation including prostate‐specific antigen progression‐free survival (PSA‐PFS) and overall survival (OS).

**Results:**

Of the 143 patients analyzed, HRR‐related mutations were detected in 51 patients (35.7%). The most frequently mutated genes were *CDK12* (*N* = 19, 13.3%), followed by *BRCA2* (*N* = 18, 12.6%), *ATM* (*N* = 8, 5.6%), and *CHEK2* (*N* = 3, 2.1%). The most common type of first‐line therapy for mCRPC was next‐generation hormonal agents (NHA, 44.4%), followed by first‐generation antiandrogens (FGA, 30.3%), and taxanes (22.5%). Commonly prescribed first−/second‐line sequential regimens included FGA/NHA (17.6%), NHA/NHA (15.5%), and NHA/taxanes (14.1%). The median PSA‐PFS and OS for the entire cohort were 5.6 and 26.1 months, respectively. Patients carrying *BRCA1/2* mutations had numerically shorter PSA‐PFS (median 3.3 vs. 5.9 months) and OS (median 20.7 vs. 27.3 months) than those without mutations.

**Conclusions:**

In conclusion, approximately one‐third of Japanese patients with mCRPC carried mutations in HRR‐related genes in this study. The real‐world outcomes of mCRPC are poor with conventional therapy, warranting an expansion of treatment options based on genetic abnormalities of the disease.

## Graphical abstract

The real‐world outcome of Japanese patients with metastatic castration‐resistant prostate cancer (mCRPC) was poor. Patients who had at least one mutation in the 15 HRR‐related genes screened had shorter median prostate‐specific antigen progression‐free survival (PSA‐PFS) and overall survival (OS) than those without (median PSA‐PFS 5.1 vs 5.9 months; median OS 21.1 vs 29.6 months). Similarly, BRCA1/2‐mutated patients had numerically shorter median PSA‐PFS (3.3 vs 5.9 months) and median OS (20.7 vs 27.3 months) than those without BRCA1/2 mutations. These results highlight the need for an expansion of mCRPC treatment options, with consideration given to the genetic background of the disease.

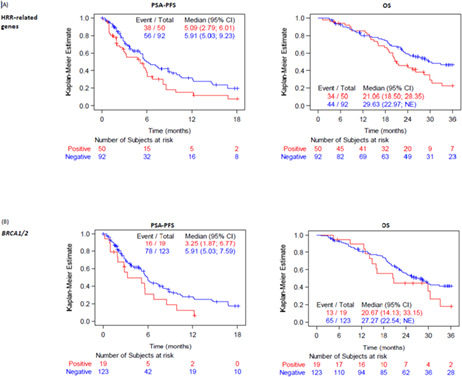

## 1 INTRODUCTION

Prostate cancer is the second most common type of cancer among men globally,^1^ with an estimated 1,414,259 new cases diagnosed in 2020.^2^ In Japan, prostate cancer is the most common cancer type in men, with 92,021 newly diagnosed cases reported in 2018.^3^

The clinical spectrum of prostate cancer varies from slow‐growing, clinically silent disease to high‐grade tumors with an aggressive course.^4^ Although patients with localized disease have an excellent outcome, outcomes of patients with metastatic castration‐resistant prostate cancer (mCRPC) are comparatively unsatisfactory when treated with conventional therapies, including next‐generation hormonal agents (e.g., abiraterone, enzalutamide) and docetaxel, with overall survival (OS) of less than 3 years.^5^, ^6^, ^7^

A growing body of evidence has elucidated the genetic heterogeneity of mCRPC,^8^, ^9^ and clinical outcomes of mCRPC may differ according to the genetic background of the disease.^10^, ^11^ Mutations are frequently detected in genes involved in the androgen receptor, cell cycle, and DNA repair (e.g., homologous recombination repair [HRR]) pathways, and some of these mutations are clinically targetable. For example, olaparib is an oral poly adenosine diphosphate (ADP)–ribose polymerase inhibitor (PARPi), currently approved globally for the treatment of *BRCA*‐mutated breast cancer, ovarian cancer, pancreatic cancer, and mCRPC. The PROfound study (NCT02987543) has shown that among patients with mCRPC carrying alterations in *BRCA1*, *BRCA2*, or *ATM*, olaparib was associated with significantly better radiographic progression‐free survival (rPFS, median 7.4 vs. 3.6 months), as well as OS (median 18.5 vs. 15.1 months), compared with control treatment (physicians' choice of either abiraterone acetate or enzalutamide).^12^ Although these data highlight the importance of identifying HRR‐related mutations to guide treatment, there is limited published information to date on the prevalence of HRR‐related gene mutations among Japanese patients with mCRPC. In this multicenter registration study titled “ZENSHIN,” we investigated the prevalence of HRR‐related gene mutations, patterns of sequential therapies, and clinical outcomes among Japanese patients with mCRPC in the real‐world setting.

## 2 METHODS

### 2.1 Study design and patients

This was a multicenter, noninterventional cohort study conducted at 24 Japanese institutions (UMIN000040511). Eligible patients were Japanese men aged ≥20 years who were diagnosed with mCRPC between January 1, 2014 and December 31, 2018 and had an archival formalin‐fixed paraffin‐embedded (FFPE) sample with formalin neutral buffer solution obtained from the primary tumor or metastatic site. All eligible patients were enrolled consecutively in order to avoid self‐selection bias. Patients who had received treatment with an investigational medicine for prostate cancer between January 1, 2014 and December 31, 2020 were excluded. Patients whose HRR‐related gene mutation testing was unsuccessful were also excluded. If the patient had died, opt‐out was applicable. Patients were followed up until December 31, 2020 or if they were lost to follow up, they were censored on the date of the last follow‐up for survival evaluation. The study was approved by an independent ethics committee (nonprofit organization MINS Institutional Review Board, Approval ID: MINS‐REC‐200208) as well as an ethics committee at each individual study site and was conducted in accordance with the Declaration of Helsinki.

### 2.2 Mutation analysis

HRR‐related gene mutation testing was performed using the archived FFPE tumor samples. Samples were tested using the Myriad myChoice HRD plus assay, which is currently approved for research use only (Myriad myChoice® HRD Plus CDx Technical Specifications). This assay detects sequence variants and large rearrangements in the following 15 HRR‐related genes: *BRCA1*, *BRCA2*, *ATM*, *BARD1*, *BRIP1*, *CDK12*, *CHEK1*, *CHEK2*, *FANCL*, *PALB2*, *PPP2R2A*, *RAD51B*, *RAD51C*, *RAD51D*, and *RAD54L*. Patients were considered to be positive for the screened mutation when pathogenic mutations were detected, whereas those without any mutations or with a variant of unknown significance (VUS) were considered to be negative.

### 2.3 Study outcomes

The primary endpoint of this study was the prevalence of tissue HRR‐related gene mutations in mCRPC patients. The secondary outcome was the proportion of treatment pattern in each line of therapy as well as the pattern of first−/second‐line sequential regimen. Treatments were categorized by drug class as next‐generation hormonal agents (NHA; e.g., abiraterone acetate, enzalutamide), first‐generation antiandrogens (FGA; e.g., flutamide, bicalutamide, chlormadinone acetate), taxanes (e.g., docetaxel, cabazitaxel), or others. Exploratory outcomes were the prostate‐specific antigen (PSA) response, which was defined as a ≥50% reduction in PSA level from the level at the start of each treatment line (PSA50 response), and survival outcomes such as PSA‐PFS and OS; exploratory outcomes were analyzed for the entire cohort as well as subcohorts stratified by the status of gene mutations and/or treatment pattern. PSA‐PFS was calculated from the start of first‐line treatment to the date of PSA progression, defined as an increase in PSA levels of ≥25% and ≥2.0 ng/ml from nadir level, death, or last follow‐up. OS was calculated from the date of mCRPC diagnosis to the date of death or last follow‐up.

### 2.4 Statistical analysis

Categorical variables were summarized by frequency and percentage and continuous variables by median and range. The Kaplan–Meier method was used to estimate median survival time. No data imputation was conducted; unavailable data were categorized as missing using descriptive statistics.

## 3 RESULTS

### 3.1 Patient characteristics

**FIGURE 1:**
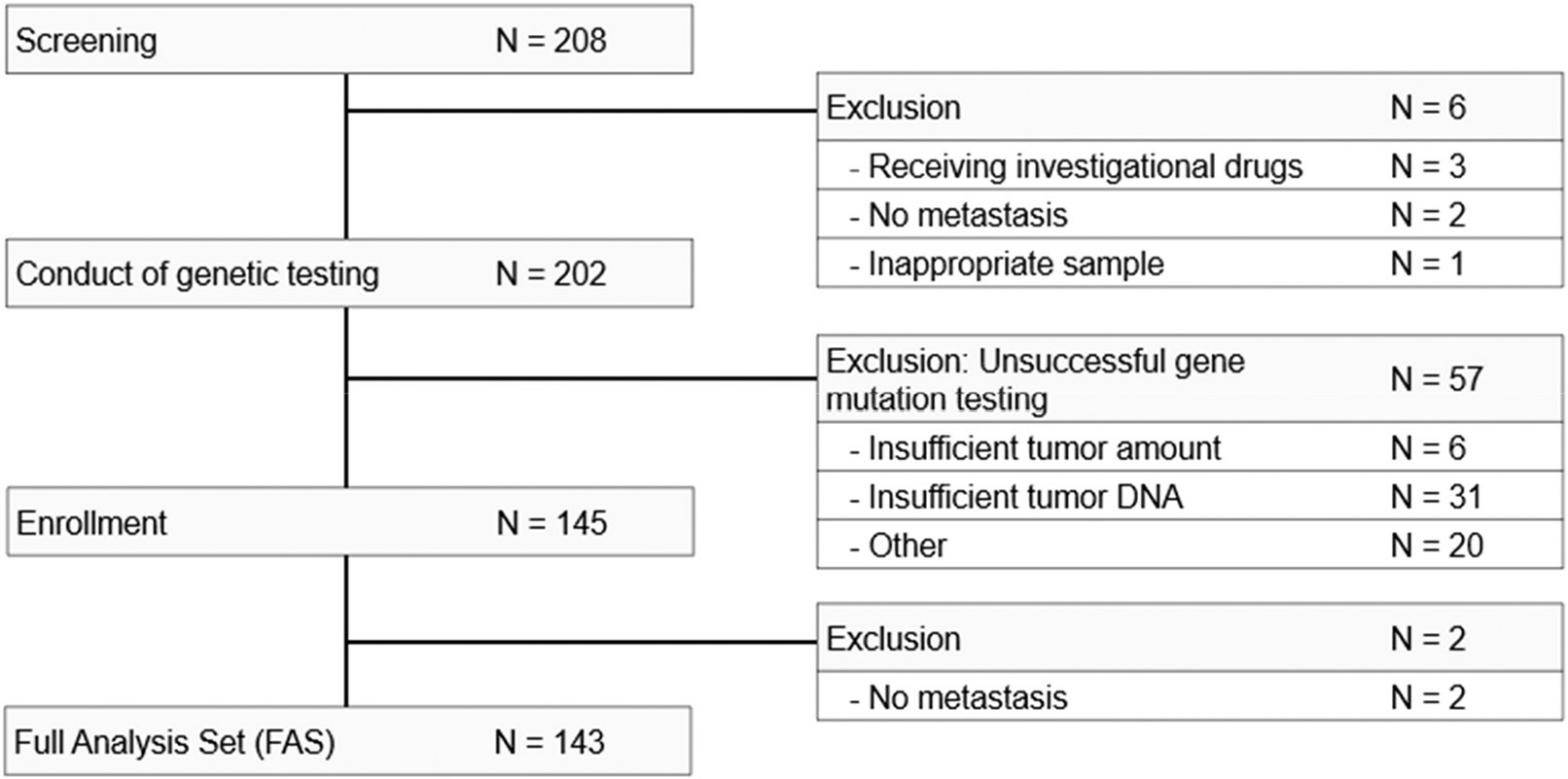
CONSORT diagram for the study population

Overall, 208 patients were screened for inclusion, of whom 145 (69.7%) had gene mutation data available. The most common reason for test failure was insufficient tumor DNA (*N* = 31). The full analysis set (FAS) included 143 patients (Figure 1). Of the 143 patients, 39 patients (27.3%) had previously participated in the PROfound study and had been randomized to the control arm, that is, had not received the investigational drug.^12^

Patient characteristics at the time of mCRPC diagnosis are summarized in Table 1. The median (range) age was 73 (52–90) years. Eleven patients (7.7%) had comorbid cancer. Seven out of 111 patients (6.3%) with available data had a family history of cancer. The most common metastatic site was bone (*N* = 123, 86.0%), followed by lymph nodes (*N* = 78, 54.5%), lung (*N* = 18, 12.6%), and liver (*N* = 7, 4.9%).

**TABLE 1:** Demographics of patients at the time of metastatic castration‐resistant prostate cancer diagnosis. Data are N (%) unless otherwise specified

	Full‐analysis set (*N* = 143)
Median age, years (range)	73.0 (52.0–90.0)
ECOG PS	
0	91 (83.5)
1	14 (12.8)
2	3 (2.8)
3	0
4	1 (0.9)
Missing data	34
Any comorbid cancer	
Yes	11 (7.7)
No	132 (92.3)
Any family history of cancer^a^	
Yes	7 (6.3)
Prostate cancer	4
Breast cancer	1
Ovarian cancer	1
Pancreatic cancer	1
No	104 (93.7)
Missing data	32
Metastatic sites^b^	
Bone	123 (86.0)
Lymph nodes	78 (54.5)
Lung	18 (12.6)
Liver	7 (4.9)
Other	4 (2.8)
Missing data	0

^a^ Up to third‐degree relative.

^b^ Patients may have >1 metastatic site.

Abbreviation: ECOG PS, Eastern Cooperative Oncology Group performance status.

Most patients had shown aggressive disease features at the time of initial prostate cancer diagnosis, including high PSA level (median 197 ng/ml, range 0.0–16,641 ng/ml) and a high Gleason score (≥8 in 132/139 patients [95%]) (Figure S1).

Patients had received the following treatments prior to their mCRPC diagnosis: androgen deprivation therapy (ADT) + FGA (*N* = 107, 74.8%), ADT alone (*N* = 24, 16.8%), ADT + docetaxel (*N* = 1, 0.7%), and other (*N* = 11, 7.7%). Definitive radiotherapy and radical prostatectomy had been performed in eight (5.6%) and seven (4.9%) patients, respectively.

### 3.2 Landscape of HRR‐related gene mutations in mCRPC

**FIGURE 2:**
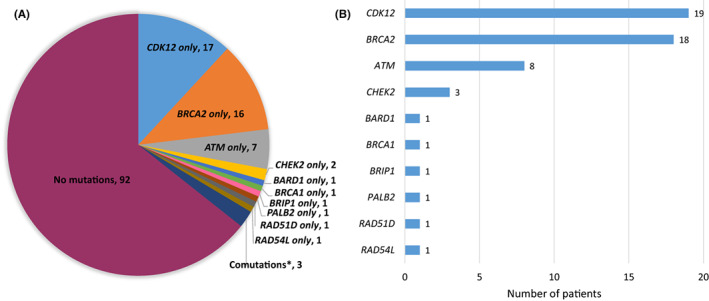
Landscape of HRR‐related gene mutations in mCRPC. (A) Frequency of mutations for the individual 15 HRR‐related genes. *Three patients had ≥1 mutation (*ATM* + *CDK12* in one patient, *BRCA2* + *CDK12* in one, and *BRCA2* + *CHEK2* in one). (B) Number of patients with each individual gene mutation. The x‐axis represents the number of patients with each gene mutation. HRR, homologous‐related repair; mCRPC, metastatic castration‐resistant prostate cancer

Overall, 51/143 patients (35.7%) carried ≥1 mutation (i.e., germline and/or somatic) in any of the 15 HRR‐related genes screened. Of the 51 patients with ≥1 mutation, 48 patients had only one mutation, whereas three patients had coexisting mutations (*BRCA2* and *CDK12*, *BRCA2* and *CHEK2*, and *ATM* and *CDK12*; *N* = 1 for each) (Figure 2A). Mutations were most commonly detected in *CDK12* (*N* = 19, 13.3%), followed by *BRCA2* (*N* = 18, 12.6%), *ATM* (*N* = 8, 5.6%), and *CHEK2* (*N* = 3, 2.1%) (Figure 2B). *BRCA1* or *BRCA2* (*BRCA1/2*) mutations were detected in 19 patients (13.3%) including one patient (0.7%) with *BRCA1* and 18 patients (12.6%) with a *BRCA2* mutation. Seven patients (4.9%) had VUS in *BRCA1/2* genes.

*BRCA1‐/2*‐mutated patients were numerically younger at the time of mCRPC diagnosis (median age 71 vs. 74 years) and had a numerically higher rate of positive family history (18.2% vs. 5.0%), compared with those without *BRCA1/2* mutations. The proportion of patients with a Gleason score of 8 including pattern 5 or higher was 78.9% versus 72.5% in *BCRA1‐/2*‐positive and *BRCA1‐/2*‐negative patients, respectively. Lymph node metastasis was observed in 78.9% and 50.8% of patients with or without *BRCA1/2* mutations, respectively. The proportion of patients who had bone metastasis as the only metastatic site was numerically lower in those with *BRCA1/2* mutations than without (15.8% vs. 40.3%). Patients with *CDK12* mutations were numerically more likely to present with higher Gleason score (8 including pattern 5 or higher, 94.4% vs. 70.2%) than those without *CDK12* mutations, but with no apparent differences between these groups in age at mCRPC diagnosis (71 vs. 73 years), rate of positive family history (6.7% vs. 6.3%), and the site of metastasis (lymph node metastasis 63.2% vs. 53.2%; bone metastasis without other metastasis 31.6% vs. 37.9%).

### 3.3 Treatment patterns

Overall, 142, 114, and 85 patients received first‐, second‐, and third‐line therapy, respectively. The most common type of first‐line therapy was NHA (*N* = 63, 44.4%), followed by FGA (*N* = 43, 30.3%), and taxanes (*N* = 32, 22.5%). NHA was also the most commonly used agent for second‐line therapy, being used in 59/114 patients (51.8%) followed by taxanes in 39/114 (34.2%). Taxanes were the most common third‐line treatment (39/85, 45.9%) and NHA was the second most common (36/85, 42.4%) (Table 2). Radiotherapy was applied in eight patients (5.6%) as first‐line treatment, in 11 (9.6%) as second‐line treatment, and in eight (9.4%) as third‐line treatment.

**TABLE 2:** Treatment patterns for metastatic castration‐resistant prostate cancer

Treatments used at each line of therapy, N (%)	Full analysis set (*N* = 143)		
	First‐line (*N* = 142)	Second‐line (*N* = 114)	Third‐line (*N* = 85)
Next‐generation hormonal agents^a^	63 (44.4)	59 (51.8)	36 (42.4)
Taxanes^b^	32 (22.5)	39 (34.2)	39 (45.9)
First‐generation antiandrogens^c^	43 (30.3)	4 (3.5)	3 (3.5)
Other	11 (7.7)	16 (14.0)	14 (16.5)

^a^ Includes abiraterone acetate and enzalutamide.

^b^ Includes docetaxel, paclitaxel, and cabazitaxel.

^c^ Includes flutamide, bicalutamide, and chlormadinone acetate.

The most common sequence of treatment used in the first‐ then second‐line setting was FGA/NHA (*N* = 25, 17.6%), followed by NHA/NHA (*N* = 22, 15.5%), NHA/taxane (*N* = 20, 14.1%), NHA/no second‐line treatment (*N* = 14, 9.9%), and taxane/NHA (*N* = 12, 8.5%) (Figure S2).

### 3.4 Survival outcomes

With a median follow‐up time of 23.2 (95% confidence interval [CI] 1.2–36.1) months, 94 patients showed PSA‐progression, and 78 died. PSA50 response was obtained in 46.3%, 37.4%, and 30.3% of patients with first‐, second‐, and third‐line treatment, respectively. Among 142 patients with available follow‐up data, the median PSA‐PFS and OS were 5.6 (95% CI 4.7–6.8) months and 26.1 (95% CI 22.1–30.5) months, respectively (Figure S3).

**FIGURE 3:**
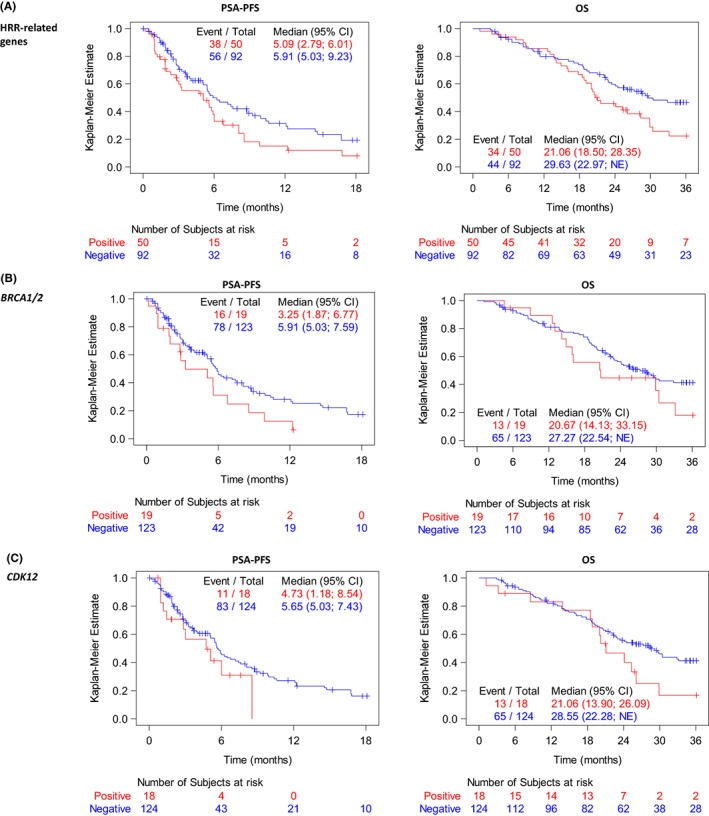
Outcome of patients with mCRPC according to gene mutation status. PSA‐PFS (left) and OS (right) estimated using the Kaplan–Meier method are shown according to the status of (A) HRR‐related gene mutations, (B) *BRCA1/2* mutations, and (C) *CDK12* mutations. The x‐axis represents time (months), and the y‐axis represents the survival rate. CI, confidence interval; HRR, homologous recombination repair; mCRPC, metastatic castration‐resistant prostate cancer; NE, not estimable; OS, overall survival; PSA‐PFS, prostate‐specific antigen‐progression‐free survival

Median PSA‐PFS was 5.1 (95% CI 2.8–6.0) months in patients who had ≥1 mutation in the 15 HRR‐related genes compared with 5.9 (95% CI 5.0–9.2) months in those who did not have any mutations, and median OS in these groups was 21.1 (95% CI 18.5–28.4) versus 29.6 (95% CI 23.0–not reached) months, respectively (Figure 3A). Similarly, patients with *BRCA1/2* mutations had a median PSA‐PFS of 3.3 (95% CI 1.9–6.8) months compared with 5.9 (95% CI 5.0–7.6) months in those without *BRCA1/2* mutations, and a median OS of 20.7 (95% CI 14.1–33.2) versus 27.3 (95% CI 22.5–not reached) months, respectively (Figure 3B). For *CDK12* mutations, the median PSA‐PFS was 4.7 (95% CI 1.2–8.5) versus 5.7 (95% CI 5.0–7.4) months with a median OS of 21.1 (95% CI 13.9–26.1) versus 28.6 (95% CI 22.3–not reached) months in patients with or without *CDK12* mutations (Figure 3C).

Median PSA‐PFS/OS by first‐line treatment was 6.5/29.2, 6.8/23.0, and 3.3/29.9 months with NHA, taxanes, and FGA, respectively (Figure S4). When patients were stratified by the type of first‐line treatment, there was no discernible difference in PSA‐PFS based on the status of *BRCA1/2* mutations, although the number of *BRCA1/2*‐positive patients in each treatment subgroup was small (Figure S5).

## 4 DISCUSSION

In this multicenter observational cohort study, we described the prevalence of HRR‐related gene mutations, as well as the treatment patterns and clinical outcomes, in Japanese patients with mCRPC in a real‐world setting. HRR‐related gene mutations were detected in 35.7% of patients, including *BRCA1/2* mutations identified in 13.3%, which was generally consistent with previous reports.^8^, ^11^, ^12^, ^13^, ^14^, ^15^, ^16^ Mutation prevalence data among Japanese patients are scarce. A study led by Momozawa et al. reported the prevalence of germline variants among Japanese patients with various stages of prostate cancer (including 8% with metastatic disease): germline *BRCA1, BRCA2*, and *ATM* mutations were detected in 0.2%, 1.1%, and 0.5% of patients, respectively.^17^ The low mutation prevalence in their study was likely due to the exclusion of somatic mutations and high proportion of early stage cases. In contrast, analysis of circulating tumor DNA obtained from 35 Japanese patients with advanced‐stage prostate cancer revealed frequent alterations in the homologous recombination deficiency pathway, including *BRCA2* mutations identified in 11% of patients, *ATM* mutations in 9%, and *CDK12* mutations in 9%.^18^ Combined with our results, although in a limited data set, these data suggest that the prevalence of *BRCA1/2* mutations may not differ between Japanese and Western populations. *BRCA2* mutations were much more frequently observed compared with *BRCA1* mutations in the current study, which was in line with previous findings.^12^, ^17^, ^19^, ^20^

The prevalence of *CDK12* mutations was high in the current study (13.3%), and almost two‐fold higher than the prevalence reported among Western populations by Wu et al. (6.9%) and de Bono et al. (7.1%).^13^, ^21^ Biallelic inactivations of *CDK12* results in a unique pattern of genomic instability are characterized by widespread focal tandem duplications and increased gene fusions.^21^, ^22^ A study by Shi et al. found that the prevalence of *CDK12* mutations was significantly higher among East Asian patients with mCRPC compared with non‐East Asians (12.9% vs. 4.2%), suggesting the presence of an ethnic disparity in mutation frequency.^23^

The 2016 guidelines from the Japanese Urological Association recommend NHA or taxanes as first‐line therapy for mCRPC but make no specific recommendations for sequential treatments.^24^ Our data showed that NHA was most commonly used in the first‐line setting (in 44.4% of patients), with relatively frequent first‐line use of FGA (in 30.3% of patients). Our study began in 2014, and since NHA became available in Japan in the same year, some of our patients may have been treated prior to the widespread use of NHA in real‐world clinical practice. Several studies have shown that individuals from Asia may respond better to ADT than those from Western countries.^25^, ^26^, ^27^ These data may have influenced the physicians' choice in selecting NHA as the first‐line treatment of mCRPC in Japanese patients.

The median OS in the current study was 26 months (about 2.2 years), which was shorter than previously reported data from clinical trials (median OS 2.5–3.0 years).^5^, ^6^, ^28^, ^29^ Although differences in patient populations and treatment regimens may account for these differences in survival, our data likely reflect the real‐world outcomes of mCRPC patients,^30^ which includes elderly patients and those with poor performance status. About 95% of patients in our study cohort had a high Gleason score (i.e., ≥8) likely due to selection bias. This may have contributed to the shorter OS we observed compared with previous reports. Our data demonstrate that patients with mCRPC have poor outcomes when treated with conventional therapies in the real‐world setting and highlight the need for more effective therapies.

Although the prognostic implications of *BRCA* mutations in mCRPC are not fully consistent across studies^31^, ^32^, ^33^, ^34^, ^35^ and may be affected by the choice of first‐line therapy, *BRCA* mutations are generally considered to be a poor prognostic factor associated with aggressive disease features and an unfavorable clinical outcome.^36^, ^37^, ^38^ Patients from our cohort carrying *BRCA1/2* mutations showed numerically shorter PFS and OS as well as worse Gleason scores. In the absence of statistical comparison, our study does not provide definitive conclusions regarding whether patients with mCRPC carrying *BRCA1/2* and *CDK12* mutations exhibit a poorer prognosis than those without such mutations, although this does not exclude the possibility that targeting these mutations may be clinically beneficial. In the PROfound study, olaparib was associated with a statistically significant 51% decrease in the risk of progression or death in the overall patient group, but an exploratory analysis showed a 78% decrease in patients with a *BRCA1/2* mutation,^12^ indicating that these mutations may be a predictive biomarker for patients who will respond favorably to PARPi therapy. Although the current indication of olaparib for mCRPC is restricted to *BRCA1‐/2*‐mutated cases in Japan, future prospective studies are expected to further elucidate the role of HRR‐related mutations beyond *BRCA1/2*, with more detailed classification (e.g., germline vs. somatic and allele frequency), as predictive biomarkers to guide the selection of the most appropriate first‐line therapy for mCRPC.

In our study, gene mutation testing was successful in approximately 70% of patients, with the main reason for failure being insufficient tumor DNA. This is comparable to the success rate in the PROfound study (69%)^12^ and indicates that there is room for improvement in sample collection and preparation.^39^ The development and implementation of liquid biopsy techniques may help to overcome some of the problems associated with tumor biomarker analysis.^40^, ^41^, ^42^

Our study has some limitations, including those inherent to a study of this nature, for example, the potential for selection bias, and random or nonrandom variable misclassification during data collection. We used only tumor samples for the mutation analysis and therefore were unable to distinguish between somatic and germline mutations; nevertheless, this approach reflects real‐world mutation screening in daily clinical practice. Approximately 70% of the samples in our study could be evaluated for gene mutations, and it is not possible to determine how the findings would differ if a higher proportion of patients had evaluable mutation information available.^15^

## 5 CONCLUSIONS

HRR‐related mutations, such as *BRCA2* and *CDK12* mutations, were frequently identified among Japanese men with mCRPC. Our results highlight the poor clinical outcomes for patients with mCRPC treated in the pre‐PARPi era and underscore the need for a wider variety of treatment options that are based on the genetic background of the disease.

## AUTHOR CONTRIBUTIONS

**Hiroji Uemura:** Conceptualization (equal); data curation (equal); resources (equal); writing – original draft (equal); writing – review and editing (equal). **Mototsugu Oya:** Conceptualization (equal); data curation (equal); resources (equal); writing – review and editing (equal). **Toshiyuki Kamoto:** Conceptualization (equal); data curation (equal); resources (equal); writing – review and editing (equal). **Mikio Sugimoto:** Conceptualization (equal); data curation (equal); resources (equal); writing – review and editing (equal). **Kenta Shinozaki:** Conceptualization (equal); data curation (equal); writing – review and editing (equal). **Kiyomi Morita:** Conceptualization (equal); data curation (equal); writing – original draft (equal); writing – review and editing (equal). **Ryo Koto:** Conceptualization (equal); data curation (equal); writing – review and editing (equal). **Mai Takahashi:** Conceptualization (equal); data curation (equal); writing – review and editing (equal). **Masahiro Nii:** Conceptualization (equal); data curation (equal); writing – review and editing (equal). **Eisei Shin:** Conceptualization (equal); data curation (equal); writing – review and editing (equal). **Norio Nonomura:** Conceptualization (equal); data curation (equal); resources (equal); writing – review and editing (equal).

## FUNDING INFORMATION

This study was supported by AstraZeneca and Merck Sharp & Dohme Corp., a subsidiary of Merck & Co., Inc., Kenilworth, NJ, USA, who are codeveloping olaparib.

## CONFLICT OF INTEREST

HU has received support for the present manuscript from AstraZeneca; has received grants from Bayer, Takeda, Daiichi Sankyo, Kyowa‐Kirin, Chugai, and Kissei; received consulting fees from AstraZeneca, Astellas, Bayer, Daiichi Sankyo, Sanofi, Chugai, Takeda, and Janssen; received honoraria for lectures from AstraZeneca, Sanofi, Bayer, Astellas, Janssen and Takeda; and has participated on advisory board of AstraZeneca, Bayer, and Daiich Sankyo. MO has received grants from Astellas; received honoraria for lectures from Astellas, AstraZeneca, Sanofi, Takeda, Janssen, Bayer, and MSD; TK has no conflict of interest/financial disclosure to declare; MS has received honoraria for lectures from Janssen, Takeda, AstraZeneca, Astellas, and Nippon Shinyaku; KS, KM, RK, MT, MN, and ES are employees of AstraZeneca K.K. and hold stocks of AstraZeneca PLC; and NN has received honoraria for lectures from AstraZeneca and Chugai.

## MEDICAL WRITING ASSISTANCE

We thank Catherine Rees of inScience Communications, Springer Healthcare, who provided writing and editorial assistance for the manuscript drafts. This medical writing assistance was funded by AstraZeneca K.K.

## ETHICS STATEMENT

The study protocol was reviewed and approved by a nonprofit organization, MINS Institutional Review Board prior to study initiation, and by the ethics committees of the participating medical institutions. The study was carried out in accordance with the Declaration of Helsinki.

## PATIENT CONSENT STATEMENT

Informed consent was obtained from all living participants. If the participant had died, opt‐out was applicable.

## Supporting information


Data S1

## Data Availability

The data that support the findings of this study are available from the corresponding author upon reasonable request.
