# Supplementary material for: The prevalence of gene mutations in homologous recombination repair pathways in Japanese patients with metastatic castration‐resistant prostate cancer in real‐world clinical practice: The multi‐institutional observational ZENSHIN study
Source: Cancer Med. 2022 Nov 10;12(5):5265–74. doi: 10.1002/cam4.5333 (PMC10028105; doi:10.1002/cam4.5333)

Supplementary Material

Supplementary Table S1. Participating physicians.

Ryuji Matsumoto (Hokkaido University Hospital)
Atsushi Takahashi (Hakodate Goryoukaku Hospital)
Naoya Masumori (Sapporo Medical University Hospital)
Yasuhiro Hashimoto (Hirosaki University Hospital)
Takayuki Yoshino (University of Tsukuba Hospital)
Jun Miki (The Jikei University Kashiwa Hospital)
Hideki Takeshita (Saitama Medical Center)
Yusuke Ito (Yokohama City University Hospital)
Yasuhide Miyoshi (Yokohama City University Medical Center)
Atsushi Mizokami (Kanazawa University Hospital)
Masashi Kato (Nagoya University Hospital)
Taku Naiki (Nagoya City University Hospital)
Keita Nakane (Gifu University Hospital)
Satoshi Anai (Nara Medical University Hospital)
Kazutoshi Fujita (Kindai University Hospital)
Tomoaki Terakawa (Kobe University Hospital)
Mutsushi Kawakita (Kobe City Medical Center General Hospital)
Atsushi Takamoto (Okayama University Hospital)
Katsuya Hikita (Tottori University Hospital)
Masanobu Shigeta (National Hospital Organization Kure Medical Center and Chugoku Cancer Center)
Hiroaki Matsumoto (Yamaguchi University Hospital)

Takuma Kato (Kagawa University Hospital)
Tadahiko Kikugawa (Ehime University Hospital)
Naoki Terada (Miyazaki University Hospital)

Supplementary Figure S1. Tumor characteristics at the time of initial prostate cancer diagnosis, (**a**) T classification; (**b**) N classification; (**c**) M classification, and (**d**) Gleason score.

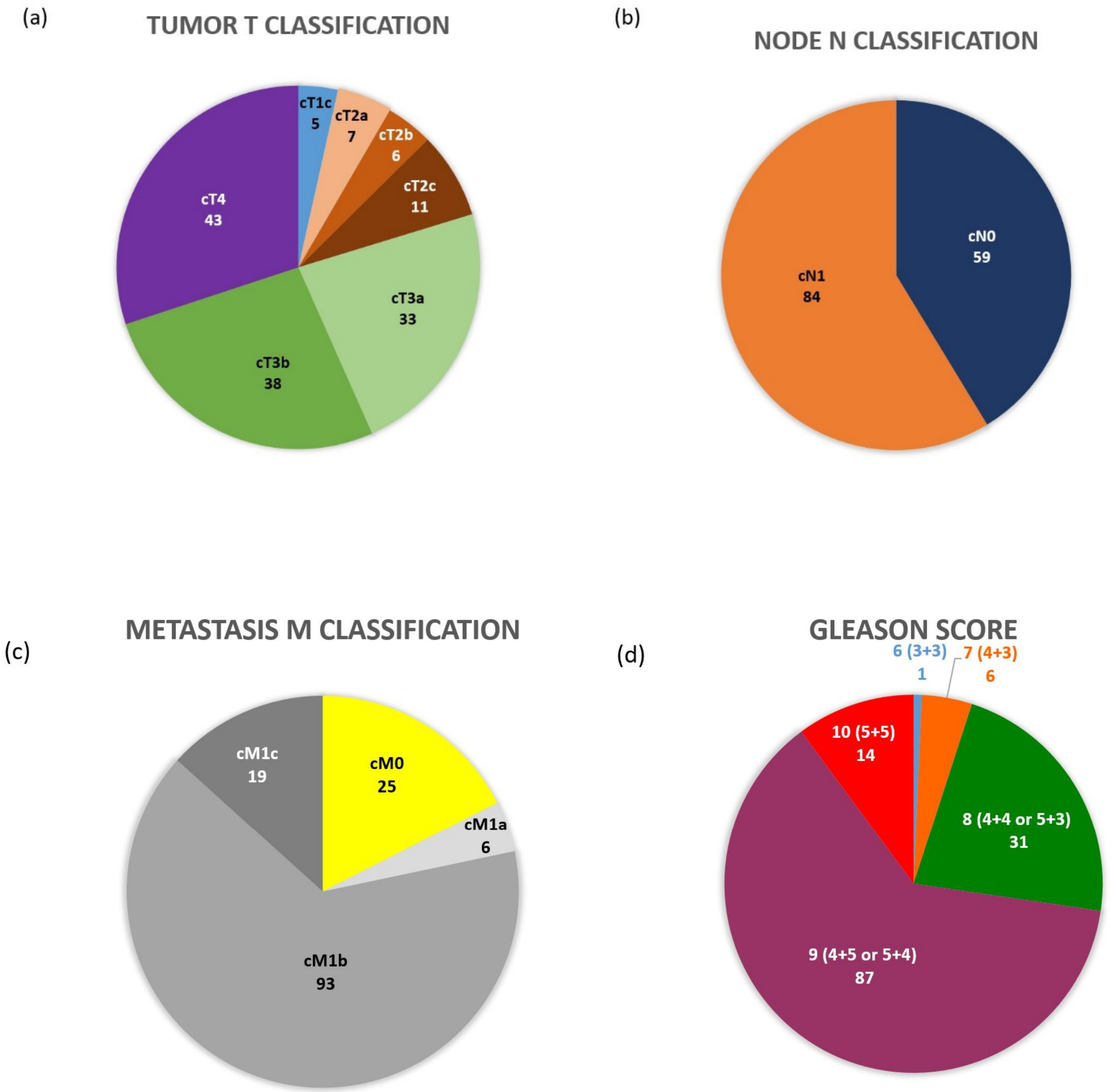

Supplementary Figure S2. Landscape of first- to second-line sequential treatment regimen. The y-axis represents the top five most frequent first- to second-line sequential therapy regimens and the x-axis represents the percentage of patients receiving each sequence.

2L, second-line; FGA, first-generation antiandrogens; NHA, next-generation hormonal agents.

Other first- to second-line sequences included: NHA → FGA (n=2, 1.4%), NHA → other (n=8, 5.6%), taxane → taxane (n=9, 6.3%), taxane → other (n=3, 2.1%), taxane → no 2L therapy (n=9, 6.3%), FGA → taxane (n=6, 4.2%), FGA → FGA (n=2, 1.4%), FGA → other (n=5, 3.5%), FGA → no 2L therapy (n=5, 3.5%), other → NHA (n=4, 2.8%), other → taxane (n=6, 4.2%), other → other (n=2, 1.4%), and other → no 2L therapy (n=1, 0.7%).

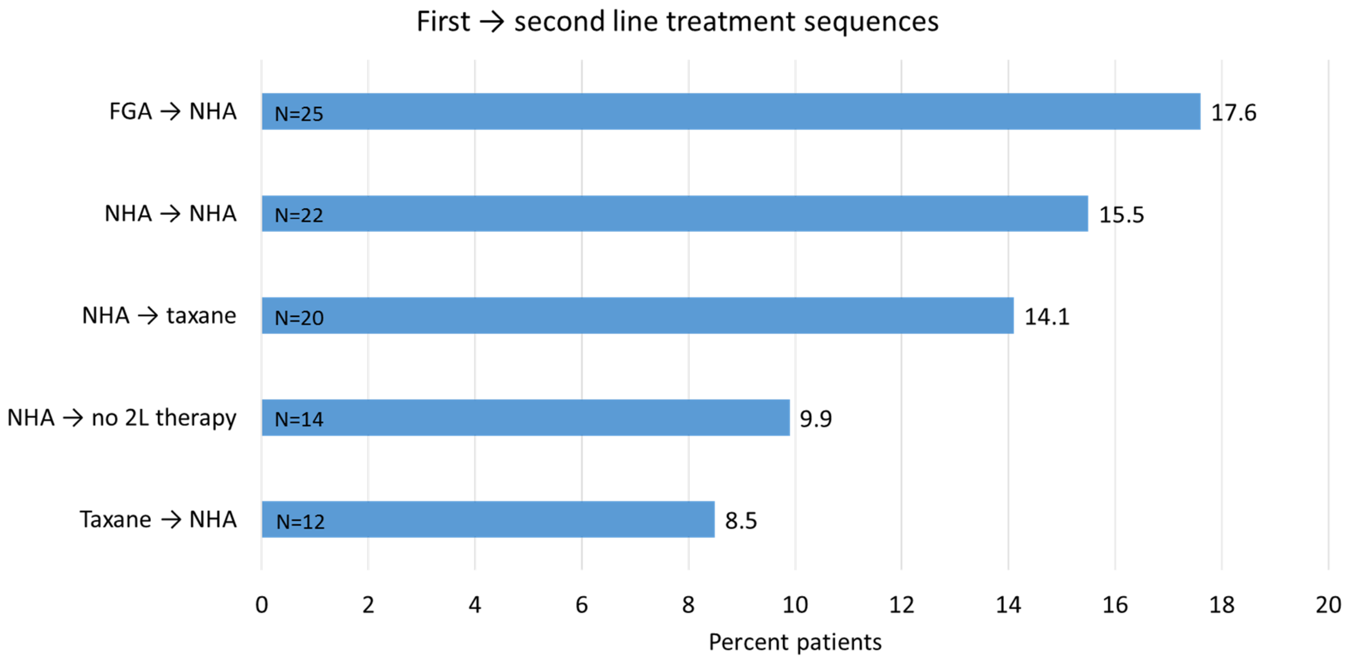

Supplementary Figure S3. Kaplan–Meier analysis of (**a**) PSA-PFS and (**b**) OS for the entire cohort. The x-axis represents time (months) and the y-axis represents the survival rate.

CI, confidence interval; OS, overall survival; PSA-PFS, prostate specific antigen progression-free survival.

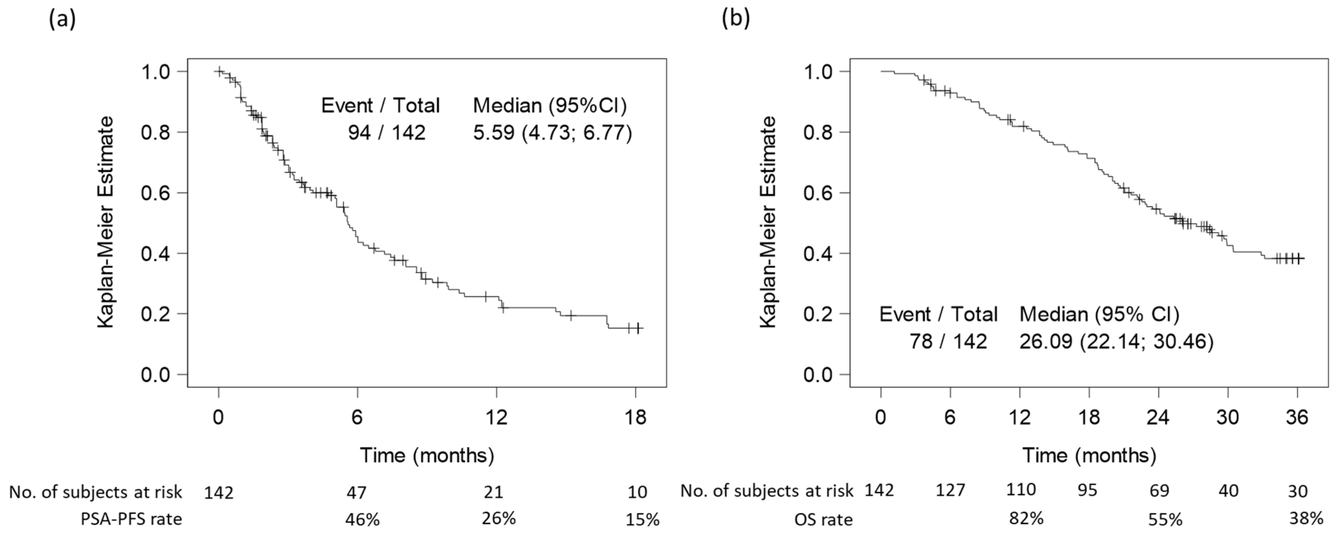

Supplementary Figure S4. Kaplan–Meier analysis of (a) PSA-PFS and (b) OS stratified by first-line treatment regimen for mCRPC. The x-axis represents time (months) and the y-axis represents the survival rate. Patients who took a combination of next-generation hormonal agents, first-generation antiandrogens, and/or taxanes were excluded from this analysis.

CI, confidence interval; FGA, first-generation antiandrogens; mCRPC, metastatic castration-resistant prostate cancer; NHA, next-generation hormonal agents; OS, overall survival; PSA-PFS, prostate-specific antigen progression-free survival.

(a)

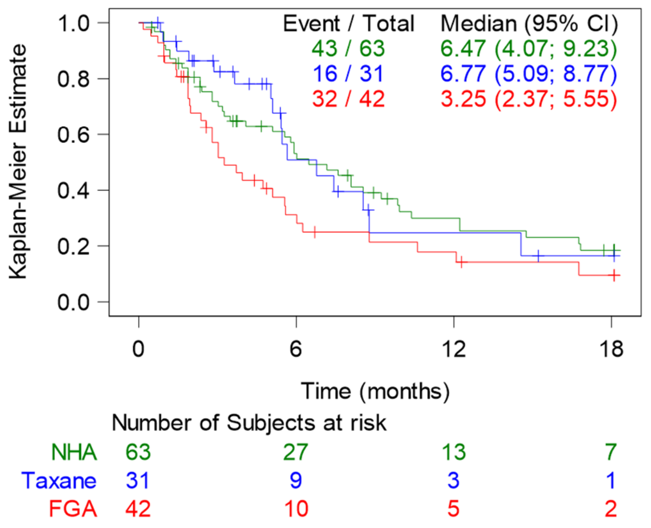

(b)

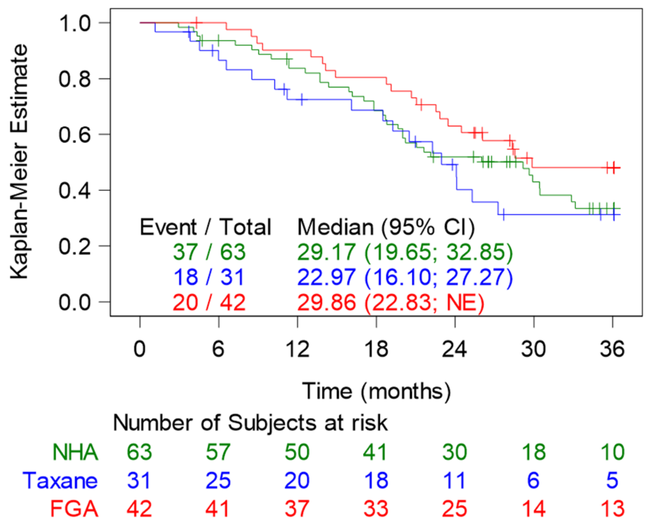

Supplementary Figure S5. Kaplan–Meier analysis of PSA-PFS from the start of first-line treatment for mCRPC, stratified by *BRCA* mutation status and first-line treatment regimen. The x-axis represents time (months) and the y-axis represents the survival rate. Patients who took a combination of next-generation hormonal agents, first-generation antiandrogens, and/or taxanes were excluded from this analysis.

CI, confidence interval; mCRPC, metastatic castration-resistant prostate cancer; PSA-PFS, prostate-specific antigen progression-free survival.

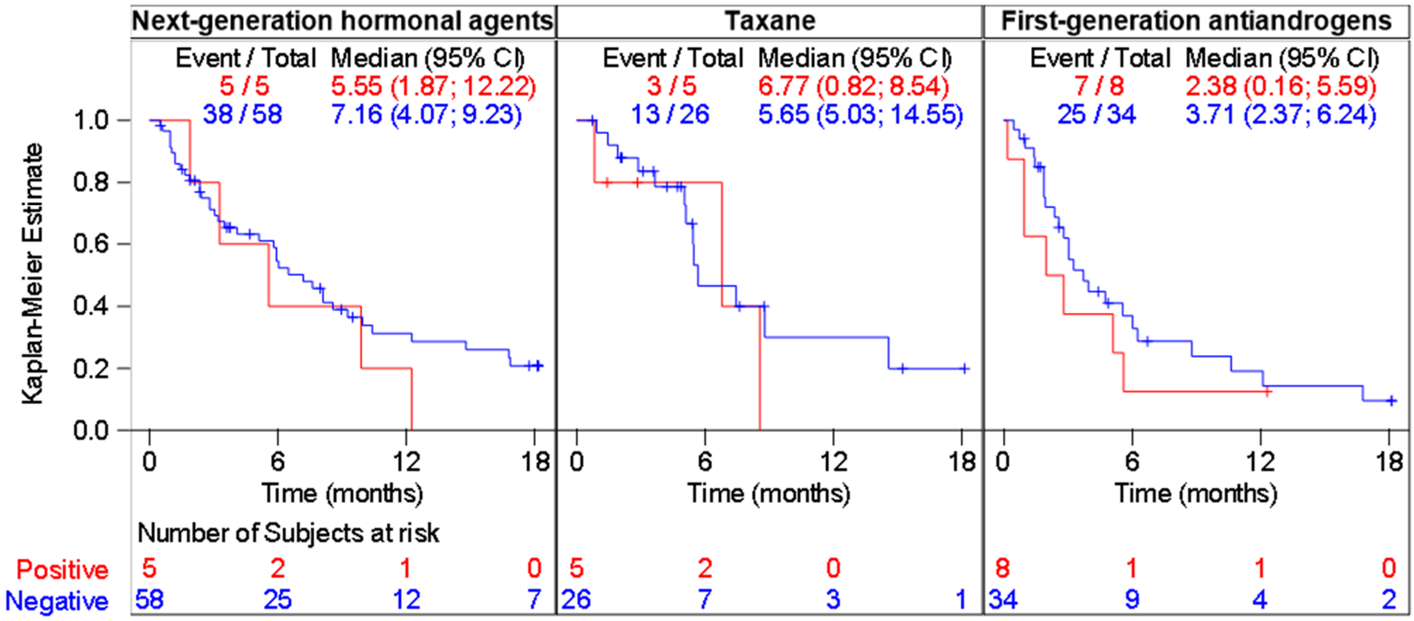

Supplement: Supplementary file 1 — Data S1 [file CAM4-12-5265-s001.pdf]
